# Prevalence and patterns of comorbidities in people with disabilities and their associated socio-demographic factors

**DOI:** 10.1038/s41598-024-51678-4

**Published:** 2024-01-16

**Authors:** Md Shohel Rana, Md Badsha Alam, Shimlin Jahan Khanam, Md Iqbal Kabir, Gulam Khandaker, Md Nuruzzaman Khan

**Affiliations:** 1 Department of Population Science, Jatiya Kabi Kazi Nazrul Islam University, Trishal, Mymensingh, Bangladesh; 2 Climate Change and Health Promotion Unit (CCHPU), Health Services Division, Ministry of Health and Family Welfare, Topkhana Road, Dhaka, 1000 Bangladesh; 3 Department of Disaster Science and Climate Resilience, University of Dhaka, Dhaka, 1000 Bangladesh; 4 Central Queensland Public Health Unit, Central Queensland Hospital and Health Service, Rockhampton, Australia; 5 School of Health, Medical and Applied Sciences, Central Queensland University, Rockhampton, Australia

**Keywords:** Medical ethics, Nutrition, Paediatrics, Public health, Diseases, Health care, Health occupations

## Abstract

The presence of comorbidities among individuals with disabilities worsens their already complex health and social circumstances. This study aims to explore prevalence and patterns of morbidities among persons with disabilities in Bangladesh and identify associated socio-demographic factors. Data from 4270 persons with disability was analysed extracted from the 2021 Bangladesh National Household Survey on Persons with Disability. Outcome variable considered was the occurrence of morbidity among persons with disabilities. Explanatory variables encompassed factors at the individual, household, and community levels. Adjusted and unadjusted multilevel mixed-effects logistic regression model was used to explore association of outcome variable with explanatory variables. We found that approximately half of individuals with disabilities experienced one or more morbidities, with chronic conditions being the most prevalent (44%). Around 42% of total persons with disability were unable to work. Specifically, hypertension (18.3%), diabetes (9.1%), and heart problems (17.1%) were prevalent chronic conditions. The likelihood of experiencing comorbidity was found to be higher among females (aOR 1.3, 95% CI 1.1, 1.7), increase year of education (aOR, 1.1, 95% CI 1.0–1.2), and those from wealthier households (aOR 1.6, 95% CI 1.2, 2.2). This underscores the need for targeted policies and interventions addressing their distinct healthcare needs.

## Introduction

Disability stands as a pervasive global concern, affecting a population of over 1 billion individuals, which is equivalent to approximately 1 in 8 people worldwide^1^. Over 80% of these population live in low- and middle-income countries (LMICs), with the rate is further rising rapidly because of population growth, increased child survival, conflicts and climate change^1–3^. The person with disability in LMICs often finds themselves marginalized from mainstream society, grappling with limited access to essential healthcare services and educational opportunities^4,5^. This situation is exacerbated by socio-economic disparities, prevailing negative cultural attitudes toward disability, and insufficient governmental support systems, all of which collectively hinder the full integration of person with disability into broader societal structures^1,3^.

Persons with disabilities are often perceived as a burden at the societal level, relying primarily on their families to meet basic needs. The majority of individuals with disabilities also depend on livelihood support offered by governmental and non-governmental organizations. This dependence may result in reduced access to healthcare services. The presence of morbidities among persons with disabilities can further intensify the complex network of health and social challenges they confront^6,7^. This is primarily attributed to their increased need for continuous access to healthcare services and the associated financial burdens, compounded by their ongoing reliance on livelihood support^8,9^. These dynamics collectively indicate a pathway towards poorer health conditions, underscoring the interconnected challenges faced by individuals with disabilities in both the health and socio-economic domains.

However, despite these critical concerns, the extent to which morbidities prevail among disabled individuals remains largely unexplored in LMICs^10,11^. Existing studies focusing on disability in LMICs have primarily centred around the prevalence of disability itself and the socio-demographic factors intertwined with it^12,13^. This deficiency in addressing the intersection of disabilities and additional health conditions has hindered governments and relevant stakeholders from accurately identifying and addressing the genuine needs of person with disability^14^. This dearth of comprehensive research is particularly disconcerting for the person with disability in Bangladesh, where the number of individuals living with disabilities surpasses nearly 1.43 million^14–16^. A majority of them are living in extreme poverty, lack education, and heavily rely on government assistance for sustenance^17,18^. To bridge this critical knowledge gap, the present study endeavours to shed light on the prevalence of morbidities among persons with disabilities and its associated socio-demographic factors.

## Methods

### Data source and sampling strategy

Data for this study were extracted from the Bangladesh National Household Survey on Persons with Disability (NSPD) 2021. This is a nationally representative cross-sectional survey conducted by the Bangladesh Bureau of Statistics. The survey followed a two-stage stratified random sampling method to select the respondents. In the first stage of sampling, 800 primary sampling units (PSUs) were randomly selected from the list of 293,579 PSUs. The PSU was generated by the Bangladesh Bureau of Statistics as a component of the 2011 Bangladesh National Population Census, derived from an average of 120 households^19^. In the second stage of sampling, 45 households were selected from each of the earlier selected PSUs through systematic random sample methods. This yields a list of 36,000 households, of them data were collected from 35,493 households. All respondents who are usual residents of the selected households were included in the survey. This covered a total of 14,659 children aged 0–4 years, 39,513 children aged 5–17 years, and 100,853 adults aged 18–95 years. Details of the sampling procedure of the surveys have been published elsewhere^16^.

### Analysed sample

This study specifically targeted individuals aged 2 and older (excluding those under 2, as identifying the need for assistive instruments or detecting disabilities is notably challenging in that age group in Bangladesh). We focused on individuals with various types of disabilities as intended. Out of all participants in the survey, a total of 4270 individuals reported having a disability, and they were included in our analysis.

### Outcome variable

Our primary objective was to investigate the occurrence of morbidities (presence of illness or health conditions) among individuals with disabilities (functional limitations or restrictions in performing activities due to impairments). Relevant data were gathered by posing two independent sets of questions. Initially, questions aimed at determining disability status were administered, followed by inquiries concerning morbidity status. To accomplish this, data collectors meticulously assessed the status of all household members, inquiring whether any reported members utilized assistive instruments, such as hearing aids, to lead a normal life. The identified respondents or their actual caregivers (for children under 18 years of age) were then subjected to a series of questions to ascertain whether the reported difficulty qualified as a disability. The Washington Group Questions for child and adult were employed for this purpose. The selected group of respondents or their actual caregivers were also presented with two additional questions to determine the presence of existing morbidities. Firstly, they were asked, "*Do you (or the name of children under 18 selected for disability-related questions) have any other health or physical problems besides your disability?*" Those responding affirmatively to this initial question were subsequently queried, "*Which type of health-related or physical health problems do you have?"* A comprehensive list of morbidities, including conditions such as blood pressure, diabetes, asthma, epilepsy, heart problems, physical/movement problems, and other health and physical issues, was provided for respondents to choose from. An option was also given to indicate if the health condition was not listed. Using these responses, we formulated a straightforward classification to discern whether a person with a disability had a morbidity or not and considered as the outcome variable.

### Explanatory variables

Several explanatory variables were included. They were selected in three stages. In the first stage, we generated a list of relevant variables through compressively reviewing relevant literature covering LMICs and Bangladesh^9,10,20–22^. The availability of these selected variables was then checked in the survey during the second stage. Finally, the available variables were considered in this study and classified as individual, household, and community-level factors. Individual-level factors include the age of the respondents (children aged 2–17, adults aged 18–34, late adult aged 35–59, older individuals aged 60 or more), gender (male vs. female), respondents’ employment status (agriculture, physical workers, business, service, students, housewives, unable to work, and others), educational attainment (no education, primary, secondary or higher), religion (muslim vs. others), and marital status (married, unmarried, and widow/divorced/separated). Household wealth status (poorest, poorer, middle, richer, richest) was considered as household-level factors. The household wealth status was created by the survey authority using principal component analysis, considering several variables related to household wealth, such as ownership of a radio or television and household roof types. Place of residence (rural vs. urban) and administrative division (Barishal, Chattogram, Dhaka, Khulna, Mymensingh, Rajshahi, Rangpur, Sylhet) were included as community-level factors.

### Statistical analysis

Descriptive statistics were used to explore the background characteristics of the respondents. Pearson chi-square test was used to identify the significant differences of the occurrence morbidity among person with disability with explanatory variables at the individual, household and community level. Two level (household, cluster) multilevel logistic regression models were used to explore the associations of morbidities among persons with disability with individual, household, and community level factors. The nested structure of the NSPD data, where individuals are nested within household and households are nested within clusters, necessitated the use of multilevel modelling^23^. Previous studies have found that multilevel modelling provides comparatively better results for such structured data than simple logistic regression models^24^. Two separate models were run for children aged 2–17 and adults aged 18–95 years. Each model was adjusted for the considered explanatory variables. Multicollinearity was checked before running each model. Sampling weight were considered in all analyses. Results were recorded as adjusted odds ratios (aOR) along with their 95% confidence intervals (95% CI). All statistical analyses were performed using Stata software version 14.0 (StataCorp.org, College Station, Texas, USA). All methods are performed according to the guidelines.

### Ethical approval

The survey we analysed conducted by the Bangladesh Bureau of Statistics. Before conducting the survey, they collected the ethical approval from the Bangladesh Medical Research Counsel of the Government of Bangladesh and from their own internal review broad. Informed consent was obtained from all the respondents before conducting interviews. When respondents were unable to provide informed consent, we obtained it from their legally authorized representatives, including husbands of women included in the survey. The shared us the non-identifiable individual’s data for this study based on our interest to conduct this particular research. Since we only get data in non-identifiable form, we do not need any further ethical approval.

## Results

### Background characteristics of the study population

**Figure 1:**
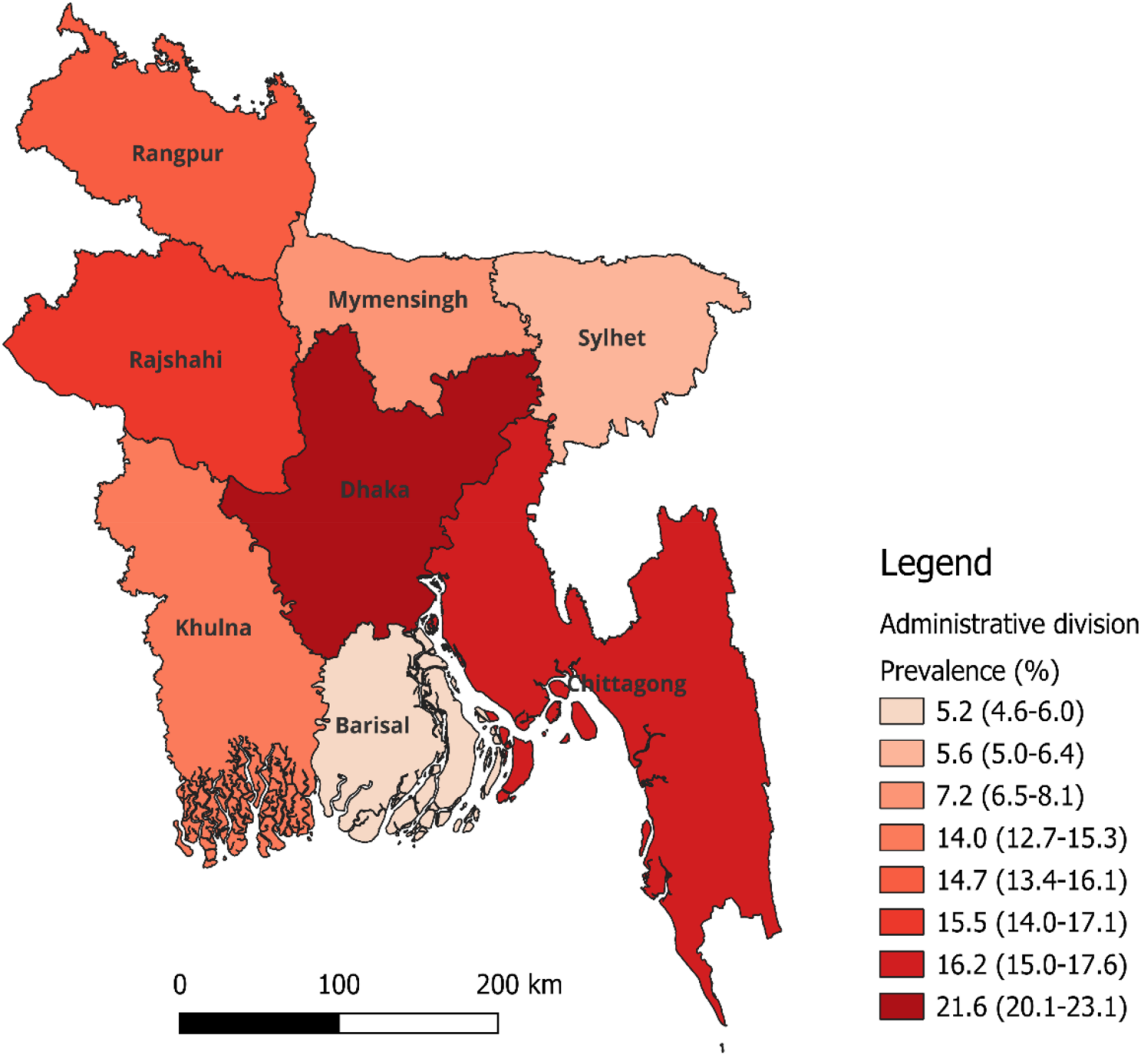
Regional distribution of persons with disability in Bangladesh.

Table 1 presents the background characteristics of the respondents. Approximately 20% of the total respondents were children aged between 2 and 17 years. Around 59% of the total respondents were male. The majority of respondents had not received any formal education (56.9%). In terms of occupation, a significant proportion of persons with disability were unable to work (41.8%), followed by housewives and students. Nearly 48% of the total respondents were married at the time of the survey. Approximately 27% of the total respondents were resided in poorest households. The majority of individuals resided in rural areas (80.7%). The largest portion of persons with disability resided in the Dhaka division (21.6%) followed by Chattogram (16.2%) and Rangpur (14.7) divisions (Fig. 1).

**Table 1:** Background characteristics of the respondents, Bangladesh, 2021, (N = 4,270).

Characteristics	Frequency	Percentage (95% CI)
Individual level factors		
Respondent’s age (in years)		
2–17	866	20.3 (19.0–21.6)
18–34	893	20.9 (19.7–22.2)
35–59	1,297	30.4 (29.0–31.8)
60 or more	1214	28.4 (27.0–30.0)
Respondent’s sex		
Male	2,502	58.6 (57.1–60.1)
Female	1,768	41.4 (39.9–42.9)
Respondent’s education		
No education	2,386	55.9 (54.2–57.5)
Primary	1,026	24.0 (22.7–25.5)
Secondary/higher	858	20.1 (18.8–21.5)
Respondent’s occupation		
Agriculture	411	9.6 (8.7–10.6)
Physical worker^+^	290	6.8 (6.0–7.6)
Business	164	3.9 (3.3–4.5)
Service	375	8.8 (7.9–9.7)
Student	484	11.3 (10.3–12.4)
Housewives	505	11.8 (10.8–12.9)
Unable to work	1,786	41.8 (40.2–43.5)
Others^++^	255	6.0 (5.2–6.8)
Respondent’s religion		
Muslim	3,822	89.5 (87.4–91.3)
Hindu and others	448	10.5 (8.4–12.6)
Respondent’s marital status		
Married	2,063	48.3 (46.7–49.9)
Unmarried	1,479	34.6 (33.0–36.3)
Widowed/divorced/separated	728	17.1 (15.9–18.1)
Household level factors		
Household wealth status		
Poorest	1,155	27.1 (25.2–29.0)
Poorer	938	22.0 (20.5–23.5)
Middle	845	19.7 (18.4–21.3)
Richer	725	17.0 (15.5–18.5)
Richest	607	14.2 (12.8–15.8)
Community level factors		
Place of residence		
Rural	3,448	80.7(79.3–82.2)
Urban	822	19.3 (17.9–20.7)
Division of residence		
Barishal	223	5.2 (4.6–6.0)
Chattogram	693	16.2 (15.0–17.6)
Dhaka	920	21.6 (20.1–23.1)
Khulna	595	14.0 (12.7–15.3)
Mymensingh	308	7.2 (6.5–8.1)
Rajshahi	661	15.5 (14.0–17.1)
Rangpur	627	14.7 (13.4–16.1)
Sylhet	243	5.6 (5.0–6.4)

All estimates are weighted. ^**+**^Physical worker includes industry or factory labour, transportation worker, day labour, rickshaw/van driver, and fisherman. ^**++**^Other occupation includes looking for work, beggar.

### Prevalence of morbidities among persons with disability in Bangladesh

Table 2 presents the percentage and types of disability and morbidities among persons with disability. Physical impairment (42.5%) was comments from of disability following by visual impairment (14.1%) and multidimensional disability (11.7%). Approximately half of the total persons with disabilities were identified as having at least one morbidity (50.0%, 95% CI: 48.4–51.8). The most prevalent types of morbidity were physical or movement problems (34.1%) following by hypertension (18.3%). Other commonest forms of morbidities were heart problems (17.1%), diabetes (9.1%), asthma (9.0%), and epilepsy (3.9%).

**Table 2:** Patterns of disability and incidence of morbidities among persons with disability in Bangladesh.

Disability	Percentages, (95% CI)	Morbidity	Percentages, (95% CI)
		Overall prevalence	50.0 (48.4–51.8)
Physical impairment	42.5 (40.8–44.2)	Physical or movement problem	34.1 (31.7–36.6)
Visual impairment	14.1 (13.1–15.3)	Hypertension	18.3 (16.5–20.3)
Multidimensional disability	11.7 (10.6–12.9)	Heart problem	17.1 (15.4–19.0)
Mental illness	8.4 (7.6–9.4)	Diabetes	9.1 (7.7–10.6)
Hearing impairment	7.0 (6.1–7.9)	Asthma	9.0 (7.8–10.3)
Intellectual disability	5.0 (4.3–5.7)	Epilepsy	3.9 (3.1–4.7)
Speech impairment	4.1 (3.5–4.8)	Other health problems	8.5 (7.3–10.1)
Cerebral palsy	2.6 (2.1–3.1)		
Autism or autism spectrum disorder	1.6 (1.2–2.0)		
Down syndrome	1.2 (0.9–1.6)		
Hearing-visual impairment	0.5 (0.3–0.7)		
Others	1.5(1.1–2.0)		

### Distribution of morbidities among persons with disability in Bangladesh across respondent’s characteristics

The percentage distribution of morbidities among persons with disability across respondent's individual, household, and community level characteristics are presented in Table 3. The prevalence of morbidities was found to be higher among older disabled persons aged 60 or more, females, persons with no formal education, those who were widowed, divorced, or separated, and those who resided in the richest households. The prevalence of morbidities varied significantly across several explanatory variables considered, except religion and place of residence.

**Table 3:** Percentage distribution of morbidities and association of the respondent’s individual, households, and community level factors.

Characteristics	Occurrence of morbidities among persons with disability		*p*-value
	No	Yes	
Overall prevalence	50.1	49.9	
Individual level factors			
Respondent’s age			
2–17	69.7	30.3	*p* < 0.001
18–34	67.4	32.6	
35–59	47.9	52.1	
60 or more	25.6	74.4	
Respondent’s gender			
Male	53.6	46.4	*p* < 0.001
Female	45.0	55.0	
Respondent’s occupation			
Agricultural workers	59.7	40.3	*p* < 0.001
Physical workers	68.5	31.5	
Business	47.1	52.9	
Services	57.2	42.8	
Students	80.3	19.7	
Housewives	43.8	56.2	
Unable to work	36.6	63.4	
Others^+^	53.9	46.1	
Respondent’s education			
No education	47.5	52.5	*p* < 0.005
Primary	53.4	46.6	
Secondary/higher	53.2	46.8	
Respondent’s religion			
Muslim	50.1	49.9	*p* = 0.857
Hindu and others	49.7	50.3	
Respondent’s marital status			
Married	43.6	56.4	*p* < 0.001
Unmarried	67.7	32.3	
Widowed/divorced/separated	32.4	67.6	
Household level factors			
Household wealth status			
Poorest	52.5	47.5	*p* < 0.01
Poorer	51.2	48.8	
Middle	50.8	49.2	
Richer	49.9	50.1	
Richest	42.9	57.1	
Community level factors			
Place of residence			
Rural	50.3	49.7	*p* = 0.517
Urban	48.9	51.1	
Division of residence			
Barishal	45.4	54.6	*p* < 0.001
Chattogram	49.2	50.8	
Dhaka	51.2	48.8	
Khulna	43.1	56.9	
Mymensingh	58.9	41.1	
Rajshahi	48.2	51.8	
Rangpur	55.0	44.0	
Sylhet	50.4	49.6	

All values are weighted and row percentage are presented. *P*-values are obtained from Pearson chi-square test. ^+^Other occupation category included looking for work, and beggar.

### Factors associated with morbidities among persons with disability in Bangladesh

The likelihoods of morbidities among persons with disability with individual, household, and community-level characteristics are presented in Table 4. We found that the likelihood of morbidities increased (aOR, 1.1, 95% CI 1.0–1.2) with the level of education among persons with disabilities. Female persons with disability reported a higher likelihood of morbidity (aOR 1.3, 95% CI 1.1–1.7) compared to male persons with disability. The likelihoods of experiencing morbidities were found lower among unmarried, divorced, and separated individuals compared to married persons with disability. Persons with disability residing in the wealthiest households reported a 1.6 times higher likelihood of morbidity (aOR 1.6, 95% CI 1.2–2.3) compared to those residing in the poorest households. We also reported lower likelihoods of morbidities among persons with disability residing in the Dhaka (aOR 0.7, 95% CI 0.5–1.0), Mymensingh (aOR 0.5, 95% CI: 0.3–0.7), and Rangpur (aOR 0.7, 95% CI 0.5–1.0) division as compared to those persons with disability residing in the Barisal division.

**Table 4:** Multilevel logistic regression model to assess the associations of morbidities of disabled persons with individual, household, and community level factors, Bangladesh, 2021.

Characteristics	Model-1 (Children aged 2–17)				Model-2 (Adult and Old aged 18–95)			
	OR (95% CI)	*p*-value	aOR (95% CI)	*p*-value	OR (95% CI)	*p*-value	aOR (95% CI)	*p*-value
Individual level factors								
Age	0.9 (0.9–1.0)	*p* < 0.01	0.9 (0.9–1.0)	*p* < 0.01	1.0 (1.0–1.0)	*p* < 0.01	1.0 (1.0–1.0)	*p* < 0.01
Gender								
Male (ref)	1.0		1.0		1.0		1.0	
Female	1.2 (0.9–1.6)	0.217	1.2 (0.9–1.7)	0.221	1.5 (1.3–1.7)	*p* < 0.01	1.3 (1.1–1.7)	*p* < 0.01
Respondents’ education	1.0 (1.0–1.0)	0.154	1.0 (1.0–1.0)	0.158	1.0 (1.0–1.0)	0.152	1.1 (1.0–1.2)	*p* < 0.01
Respondents’ occupation								
Agricultural workers (ref)	na		na		1.0		1.0	
Physical workers	na		na		0.6 (0.5–0.9)	*p* < 0.01	0.9 (0.6–1.3)	0.540
Business	na		na		1.7 (1.1–2.5)	*p* < 0.01	1.7 (1.1–2.4)	*p* < 0.05
Services	na		na		1.1 (0.8–1.5)	0.451	1.1 (0.8–1.6)	0.554
Students	na		na		0.4 (0.2–0.7)	*p* < 0.01	1.0 (0.6–1.9)	0.974
Housewife	na		na		1.8 (1.4–2.4)	*p* < 0.01	1.6 (1.1–2.3)	*p* < 0.01
Unable to work	na		na		3.1 (2.5–4.0)	*p* < 0.01	2.6 (2.0–3.4)	*p* < 0.01
Others	na		na		1.2 (0.8–1.9)	0.373	1.6 (1.0–2.6)	*p* < 0.05
Respondents’ religion								
Muslim (ref)	1.0		1.0		1.0		1.0	
Hindu and others	1.0 (0.5–1.7)	0.859	0.9 (0.5–1.6)	0.814	1.0 (0.8–1.3)	0.944	0.9 (0.7–1.1)	0.326
Respondents’ marital status								
Married (ref)	na		na		1.0		1.0	
Unmarried	na		na		0.4 (0.3–0.5)	*p* < 0.01	0.7 (0.6–1.0)	*p* < 0.01
Widowed/divorced/separated	na		na		1.6 (1.3–2.0)	*p* < 0.01	0.8 (0.6–1.0)	*p* < 0.05
Household level factors								
Household wealth status								
Poorest (ref)	1.0		1.0		1.0		1.0	
Poorer	1.0 (0.7–1.5)	0.896	1.0 (0.7–1.6)	0.884	1.0 (0.8–1.3)	0.805	1.0 (0.8–1.2)	0.812
Middle	0.8 (0.5–1.2)	0.292	0.8 (0.5–1.3)	0.408	1.1 (0.9–1.4)	0.294	1.0 (0.8–1.2)	0.861
Richer	1.0 (0.6–1.5)	0.912	1.0 (0.6–1.7)	0.942	1.1 (0.9–1.4)	0.328	1.0 (0.8–1.3)	0.853
Richest	0.8 (0.5–1.3)	0.331	0.9 (0.5–1.5)	0.640	1.9 (1.5–2.4)	*p* < 0.01	1.6 (1.2–2.3)	*p* < 0.01
Community level factors								
Place of residence								
Rural (ref)	1.0		1.0		1.0		1.0	
Urban	0.7 (0.5–1.1)	0.121	0.7 (0.4–1.1)	0.124	1.3 (1.0–1.5)	*p* < 0.05	1.0 (0.8–1.3)	0.906
Division of residence								
Barishal (ref)	1.0		1.0		1.0		1.0	
Chattogram	1.1 (0.6–2.2)	0.683	1.1 (0.6–2.1)	0.795	0.9 (0.6–1.2)	0.368	0.8 (0.6–1.2)	0.275
Dhaka	0.9 (0.5–1.7)	0.759	1.1 (0.5–2.1)	0.933	0.8 (0.6–1.1)	0.227	0.7 (0.5–1.0)	*p* < 0.05
Khulna	1.6 (0.8–3.2)	0.142	1.7 (0.9–3.5)	0.127	1.0 (0.7–1.5)	0.865	1.1 (0.7–1.6)	0.805
Mymensingh	1.2 (0.6–2.4)	0.619	1.1 (0.5–2.3)	0.756	0.5 (0.3–0.7)	*p* < 0.01	0.5 (0.3–0.7)	*p* < 0.01
Rajshahi	0.9 (0.4–1.7)	0.667	0.8 (0.4–1.6)	0.570	0.9 (0.7–1.3)	0.680	0.9 (0.6–1.4)	0.572
Rangpur	0.6 (0.3–1.3)	0.198	0.6 (0.3–1.3)	0.206	0.7 (0.5–0.9)	*p* < 0.01	0.7 (0.5–1.0)	*p* < 0.05
Sylhet	0.8 (0.4–1.6)	0.446	0.7 (0.3–1.5)	0.341	0.9 (0.6–1.3)	0.507	0.9 (0.6–1.5)	0.724

CI: Confidence interval. Ref: Reference category.

## Discussion

This study aimed to explore the prevalence of morbidities among persons with disability in Bangladesh and their connections to individual, household, and community-level factors. Our findings reveal that nearly half of all persons with disability in Bangladesh experience at least one morbidity, encompassing chronic conditions and epilepsy. The likelihood of encountering these additional morbidities among individuals with disabilities was notably higher for those with relatively higher levels of education, females, married respondents, those residing in the wealthiest households, and those living in the Dhaka, Mymensingh, and Rangpur divisions. These results are robust, given the comprehensive analysis of a large national-level sample and the consideration of a wide array of factors at the individual, household, and community levels. As such, these findings are poised to inform evidence-based policies and programs aimed at enhancing the well-being of the persons with disability in Bangladesh as well as other LMICs^25^.

The estimated prevalence of about 50% of health issues among individuals with disabilities is a cause for concern. Unfortunately, we were unable to compare this finding with the available estimates in LMICs and Bangladesh due to the lack of relevant data^6,26,27^. This higher prevalence indicates that the challenges faced by the persons with disabilities are multidimensional, encompassing issues related to both disability and morbidity, either individually or in combination^28,29^. This underscores the need for a greater role of healthcare services to support this group. However, this is particularly concerning for Bangladesh, similar to other LMICs, where healthcare facilities are mostly not disable-friendly in terms of access, compounded by the common characteristic of overcrowding^8^. The higher prevalence of disability among comparatively lower-educated and poor individuals exacerbates the situation^30^. Considering their requirement for long-term financial support, the inadequacy of such support in Bangladesh and other LMICs becomes even more pronounced. As of the recent national budget in 2023, disabled individuals receive only BDT 800 (~ 8 USD) per month^31,32^. This meagre amount of financial support is supplemented by the fact that only 4% of the total persons with disability receives financial assistance from the government, with 47.4% having received any allowances at least once in their lifetime^16^. Challenges of this nature present an insurmountable burden to achieving the Sustainable Development Goals if these individuals are excluded from the mainstream development process in Bangladesh^4^.

More than 44% of persons with disabilities have reported experiencing various chronic conditions, including diabetes (9%), hypertension (18%), and heart problems (17%). These prevalence estimates are consistent with the average rates of these conditions in Bangladesh^6,11^. However, they also highlight a growing concern due to the fact that chronic conditions often demand meticulous management and long-term care, aspects that can pose substantial challenges for people with disabilities in Bangladesh^18^. For instance, maintaining a healthy lifestyle through ensuring access to nutritious food and engaging in regular physical exercise is fraught with difficulties for this demographic^17,31^. Additionally, the financial support they receive, combined with their personal capacities, may not suffice to enable consistent access to healthcare services required for effectively managing chronic conditions^6,33^. Consequently, these dual challenges may result in untreated and unmanaged chronic conditions, potentially leading to the emergence of further health complications and premature fatalities^14^.

We observed a slight increase in the likelihood of morbidities with the rising level of education among adults with disabilities. The reasons behind these associations are mostly unknown. However, it is plausible that individuals with higher levels of education in Bangladesh may face a greater burden of chronic conditions, which often remain untreated and subsequently contribute to disability in later stages of life^34^. This is particularly noteworthy given the elevated occurrence of disability resulting from road traffic injuries among relatively higher educated individuals in Bangladesh^35^. Furthermore, higher likelihood of morbidities among persons with disability in the wealthiest households and females further bolsters this understanding. However, we could not find any logical reasoning about the divisional level variations in the likelihoods of morbidity among the persons with disability, as reported in this study. Unfortunately, we were unable to validate these findings and explore possible pathways due to the lack of available literature. As such, we recommend further studies to delve into this aspect.

This study has several strengths and a few limitations. The data analyzed in this study were extracted from a nationally representative survey with a large sample size. The estimates reported in this study are the very first of their kind in Bangladesh as well as other LMICs. The findings of this study have been adjusted for a comprehensive range of confounding factors, carefully selected through an extensive literature search and rigorous statistical analysis. Advanced statistical techniques were employed to determine the associations. Consequently, these findings will aid policy makers in formulating evidence-based policies and programs that ultimately contribute to the betterment of persons with disability. However, the primary limitations of this study lie in the analysis of cross-sectional data. Thus, the findings presented in this study are correlational only rather than causal. The survey relies on self-reported morbidity data, which could potentially result in the misreporting of certain forms of morbidities. Furthermore, the survey employs the Washington Group’s Module to assess disability, utilizing a set of questions answered by respondents or their caregivers to identify individual disabilities. The reliance on self-reported data introduces the potential for reporting bias. However, any such bias is assumed to be random, and the use of this globally recognized disability measuring tool provides evidence of the accuracy of the reported estimates. We excluded children under 2 years of age due to the potential for misreporting or undiagnosed disability status. Furthermore, we aggregated all types of disabilities into an overarching variable representing disability status. This reclassification might potentially underestimate the nuanced effects of specific disabilities. For example, mental illness and physical impairment differ significantly, and the associated morbidities in these two groups can yield distinct consequences and explanations. However, investigating these distinct effects falls outside the scope of this study. Our dichotomized reclassification of disability status aligns with global literature practices and is supported by the Washington Group's Module for assessing disability. Furthermore, various healthcare facility-level factors, including access to healthcare services and proximity to the nearest healthcare facilities, could potentially play a significant role in the occurrence of morbidity among persons with disability. These factors merit consideration in the analytical model, but their inclusion was infeasible due to the lack of relevant variables in the survey. Despite these limitations, this study furnishes a valuable understanding of the prevalence of morbidity among persons with disability in Bangladesh.

## Conclusion

Approximately 50% of persons with disabilities reported experiencing of at least one morbidity. The most prevalent types of morbidity among persons with disability were various chronic conditions, such as hypertension, diabetes, and heart problems. The likelihood of experiencing morbidity was found to be higher among those with relatively higher education levels, females, and the most affluent persons with disability. These findings underscore the necessity for enhanced support in both financial and healthcare access domains. Increasing financial allowances for persons with disability, alongside the implementation of policies and programs that ensure disability-friendly healthcare services, becomes imperative in light of these insights.

### Supplementary Information

Supplementary Information.

## Data Availability

The authors have included all the associated data related to this study in the manuscript. For any additional data pertinent to the manuscript, interested parties should reach out to the corresponding author. However, to obtain access to the complete dataset, researchers are required to contact the Bangladesh Bureau of Statistics (https://bbs.gov.bd) and submit a formal research proposal, similar to the process we followed.

## Abbreviations

LMICs: Low- and middle-income countries
NSPD: National survey on persons with disability
WHO: World Health Organizations
SDG-s: Sustainable development goals
NCD: Non-communicable disease
PHC: Primary health centres
EAs: Enumeration areas
PSU: Primary sampling unit
aOR: Adjusted Odds Ratio
CI: Confidence Interval
ref: Reference
BDT: Bangladesh Taka
